# Early detection and management of hearing loss to improve the quality of life

**DOI:** 10.2478/abm-2024-0001

**Published:** 2024-03-20

**Authors:** Editorial Office of Asian Biomedicine

**Affiliations:** Faculty of Medicine, Chulalongkorn University, Bangkok 10330, Thailand

Hearing loss in early life is an important problem because it can hinder access to spoken language, cognition, development, and social well-being [1]. There are several types of hearing loss including conductive loss (defect of the outer or middle ear), sensorineural hearing loss (SNHL) (defective inner ear structures such as outer and inner hair cells of the cochlea and the auditory neural pathway), and auditory neuropathy (AN) (defective neural processing of auditory stimuli including the eighth cranial nerve, auditory brain stem, or cerebral cortex). In many patients, hearing loss can be a combination of conductive loss, SNHL, and AN [2].

Automated auditory brainstem responses and otoacoustic emissions are routinely used as screening tests [3, 4]. Both tests are portable, automated, and inexpensive, and therefore, are commonly used for newborn screening.

Healthy newborns who pass the hearing screen should be periodically monitored for defective language acquisition, auditory skills, middle ear status, and attention to any parental-caregiver concerns [5], since infants who pass the newborn hearing screen may have risk factors for hearing loss that might be overlooked during the initial screening. These can include monitoring language milestones, routine developmental surveillance, identifying risk factors, and eliciting any parental concerns.

Children who have abnormal audiology assessments should be referred to otolaryngology, speech-language pathology, and early intervention. Referral to a clinical geneticist may be warranted if there are syndromic features or if the child has bilateral SNHL. The approach to the diagnostic evaluation is based on the type of hearing loss (sensorineural versus conductive and unilateral versus bilateral) and the age of the patient. For those with bilateral SNHL, comprehensive genetic testing is warranted.

In this issue, Jiang et al. [6] have reported the use of a reverse dot blot assay for 13 mutations of hearing loss genes in primary hospitals. This can serve as a gene detection system based on the reverse dot blotting assay technique to simultaneously detect 13 mutation sites in four hearing loss-related genes. The technique can be used in primary hospital settings with common PCR instruments. It is possible to manipulate with low cost, desirable characteristics including high performance, and high accuracy. It has a wide clinical application prospect.
